# Population Prevalence and Correlates of Syphilis in Rural, Southwestern Uganda

**DOI:** 10.1093/ofid/ofaf290

**Published:** 2025-05-28

**Authors:** Pooja Chitneni, Nicholas Musinguzi, Charles Baguma, Justin M Rasmussen, Emily N Satinsky, Justus Kananura, Patience Ayebare, Patrick Gumisiriza, Godfrey Masette, Mark J Siedner, Jessica E Haberer, Lynn T Matthews, Bernard Kakuhikire, Alexander C Tsai

**Affiliations:** Department of Global Health Equity, Brigham and Women's Hospital, Boston, Massachusetts, USA; Center for Global Health, Massachusetts General Hospital, Boston, Massachusetts, USA; Harvard Medical School, Boston, Massachusetts, USA; Mbarara University of Science and Technology, Mbarara, Uganda; Mbarara University of Science and Technology, Mbarara, Uganda; Department of Psychology and Neuroscience, Duke University, Durham, North Carolina, USA; Department of Psychology, University of Southern California, Los Angeles, California, USA; Mbarara University of Science and Technology, Mbarara, Uganda; Mbarara University of Science and Technology, Mbarara, Uganda; Mbarara University of Science and Technology, Mbarara, Uganda; Mbarara University of Science and Technology, Mbarara, Uganda; Center for Global Health, Massachusetts General Hospital, Boston, Massachusetts, USA; Harvard Medical School, Boston, Massachusetts, USA; Mbarara University of Science and Technology, Mbarara, Uganda; Center for Global Health, Massachusetts General Hospital, Boston, Massachusetts, USA; Harvard Medical School, Boston, Massachusetts, USA; Division of Infectious Diseases, Heersink School of Medicine, University of Alabama at Birmingham, Birmingham, Alabama, USA; Mbarara University of Science and Technology, Mbarara, Uganda; Center for Global Health, Massachusetts General Hospital, Boston, Massachusetts, USA; Harvard Medical School, Boston, Massachusetts, USA; Department of Epidemiology, Harvard T. H. Chan School of Public Health, Boston, Massachusetts, USA

**Keywords:** prevalence, seroprevalence, sub-Saharan Africa, syphilis, Uganda

## Abstract

**Background:**

The global incidence of syphilis has increased in recent years. Understanding syphilis epidemiology will inform screening and treatment programs. However, such data are lacking in many communities. We outline a population-based syphilis screening program in a rural community in southwestern Uganda to describe the population prevalence of syphilis.

**Methods:**

In June 2019 we conducted a cross-sectional, population-based study of adults >18 years of age. Two-stage syphilis testing was completed with *Treponema pallidum* hemagglutination (TPHA) rapid immunochromatographic testing, confirmed by rapid plasma reagin (RPR) in those with positive TPHA (syphilis seroprevalence). We calculate inverse probability of treatment (IPT) weights using logistic regression to estimate the population prevalence of positive TPHA. We included covariates with a univariable α = .10 in multivariable logistic regression models, stratified by sex, to estimate correlates of syphilis seroprevalence.

**Results:**

Among 749 participants who participated in this population-based study, 724 (97%) completed syphilis screening. The median age was 42.9 years (standard deviation, 15.6 years) and 456 of 724 (63%) were women. Based on the IPT-weighted adjusted model, the syphilis population seroprevalence was 10.6% (95% confidence interval, 8.4%–13.4%). Among the 62 of 79 (78%) participants who completed RPR testing, all had titers ≤1:4. Syphilis seroprevalence was associated with less education, human immunodeficiency virus (HIV) infection, and ≥2 sexual partners in the prior month among women and with HIV among men.

**Conclusions:**

We describe a high prevalence of current or former syphilis (10.6%) in a population-based study in rural Uganda. Syphilis screening and surveillance programs in this region require expansion to capture populations not routinely in care.

The global epidemic of syphilis (caused by the bacterium *Treponema pallidum*) has worsened in recent years, with the estimated incidence steadily rising from 8.8 million in 1990 to 14.1 million in 2019 [1]. Prior research demonstrates that settings with the highest syphilis prevalence have the lowest economic development and thus the fewest resources for syphilis screening [1, 2]. This concept is reinforced by the Spectrum-STI model (used to estimate country-specific sexually transmitted infection [STI] trends over time) that found the World Health Organization Africa region had the highest syphilis prevalence at 1.6% [2], while the global syphilis prevalence was 0.5%.

Syphilis diagnosis requires complex testing for both treponemal and nontreponemal antibodies. Traditionally, the syphilis screening algorithm tested nontreponemal antibodies followed by treponemal antibodies. More recently, the so-called reverse-syphilis screening algorithm has gained prominence due to its increased sensitivity and tests treponemal antibodies followed by nontreponemal antibodies [3]. Treponemal antibody testing (eg, *T pallidum* hemagglutination assay [TPHA] and *T pallidum* particle agglutination [TPPA]) gives a binary positive or negative result, with positive test results remaining positive for decades, if not a lifetime. This assay is widely available as a point-of-care (POC) lateral flow test [4]. After a positive treponemal antibody test, nontreponemal antibody testing (eg, rapid plasma reagin [RPR] and Venereal Disease Research Laboratory [VDRL]) is performed. Nontreponemal antibody testing conveys quantitative titers that clinicians compare over time to assess treatment outcomes. However, nontreponemal antibody testing requires increased laboratory skill and equipment. Thus, few clinical and research facilities across the world routinely incorporate this testing to determine syphilis cure, persistence, relapse, or reinfection [4].

Syphilis can cause devastating outcomes among people who do not receive a timely diagnosis and treatment. People with human immunodeficiency virus (HIV) and pregnant people are priority populations for syphilis screening given the synergy between HIV and syphilis and the poor fetal outcomes associated with syphilis, including miscarriage, stillbirth, prematurity, low birthweight, and disability [5, 6]. Additionally, syphilis can cause high morbidity among all adults, initially causing chancres and progressing to rashes, with rare instances of long-term neurological and cardiac complications [7]. Syphilis can also increase the risk of acquiring HIV and other STIs [8].

Most resource-limited settings do not allocate resources to screen adults for syphilis outside of HIV and antenatal clinics. Thus, in these settings, we have little knowledge of syphilis prevalence in general population samples. To address this gap, we conducted a cross-sectional study to estimate the population prevalence of syphilis in a rural region of southwestern Uganda. Our health screening included treponemal antibody testing (via TPHA), with positive tests confirmed by nontreponemal antibody testing (via RPR).

## METHODS

### Study Design and Setting

The study was conducted in Nyakabare parish, a rural community comprised of 8 villages 20 km from Mbarara town in southwestern Uganda. Parish populations in this region range from hundreds to thousands. In Uganda, local governments are organized by villages, with a parish comprised of several villages. The local economy of Nyakabare parish centers around subsistence agriculture, and food and water insecurity are widespread [9–11]. The health screening was nested within an ongoing population cohort study initiated in Nyakabare parish in 2014 [12]. The primary aim of the cohort study was to characterize HIV stigma in the context of social networks and understand its impacts on HIV care. All adults and emancipated minors who reported having stable, primary residence in Nyakabare parish were eligible for inclusion. There were no other exclusion criteria.

In June 2019, all adults residing in Nyakabare parish were invited to attend 1 of 5 health screening fairs in which we screened participants for various health conditions including HIV and syphilis. Those already enrolled in the cohort study and who attended the health screening were eligible for inclusion in this analysis. Based on feedback we obtained from the community leaders and a community sensitization meeting [13], we incorporated syphilis screening into the health screening fair. The health screenings were advertised through community meetings, printed advertisements, and radio and church announcements. Free transportation was arranged for community members with poor health or limited mobility [14].

### Study Procedures

Interview data were collected through structured surveys. Due to cultural norms and local laws criminalizing sexual and gender minorities, we did not elicit sexual orientation or gender. In this analysis, we assumed that people who marked their sex as female were cisgender women (henceforth referred to as “women”) and that people who marked their sex as male were cisgender men (“men”). Blood samples were collected by trained phlebotomists. Participants who agreed to participate in the syphilis screening completed screening consistent with the reverse algorithm. Participants first completed a fingerstick for POC immunochromatographic testing to detect treponemal antibodies (SD Bioline, Abbott Diagnostics, Abbott Park, IL, USA). Participants with a positive TPHA test, indicating syphilis seroprevalence, then underwent a venipuncture blood draw. Blood samples were transported from the health screening sites to the Mbarara University of Science and Technology Research Laboratory, where serum was separated for RPR testing performed at a later time. RPR testing was conducted for research purposes only, as this test is not routinely employed in most clinical settings in Uganda and therefore would not have changed clinical practice. Results were read as titers of serial dilutions (1:1, 1:2, 1:4, etc). Participants with syphilis seroprevalence were counseled on treatment and secondary transmission prevention and were offered benzathine penicillin treatment onsite. We collected participants’ contact information and linked them to a local health center for second and third doses of benzathine penicillin (corresponding with presumptive late latent syphilis treatment, per Ugandan Ministry of Health STI treatment guidelines [15]). Participants with positive results for HIV were also referred to local health centers for care.

### Outcome Measures

The primary outcome was a positive TPHA, which we refer to as syphilis seroprevalence. Covariates of interest were chosen based on the scientific plausibility of their association with syphilis seroprevalence: age, sex, education level, household asset wealth [16, 17], STI symptoms/syndromes, number of sexual partners, experience of intimate partner violence [18], and HIV serostatus (both self-reported and newly diagnosed via POC testing onsite at the health screening).

### Analysis

Attendance at the health screening fairs was voluntary, so syphilis seroprevalence estimates based on attendees might not reflect the population seroprevalence in the region. Because the health screening fairs were embedded within a larger parent study that collected data on nearly the entire population (response rate 98% at 2014 cohort initiation)—both attendees and nonattendees—we used inverse probability of treatment (IPT) weights to estimate population prevalence. We first estimated the propensity to participate in the health screening using a logistic regression model consisting of 14 variables expected to impact health screening attendance: age, sex, education level, marital status, village of residence, household asset wealth [16], distance from the health screening site, self-reported overall health [19] (a 4-point Likert-type scale ranging from very good to very bad health), self-reported HIV status, heavy alcohol use, social network size [12, 20], index of social participation [21], water insecurity [11], and food insecurity [22]. We used the following definitions: (1) social network size was determined as the total number of unique social network ties elicited in response to a series of culturally adapted social network name generator questions (informally understood as a participant's total number of “friends”) [12, 20]; (2) social participation was the number of different community groups in which the participant reported significant participation in the previous 2 months [21]; and (3) water and/or food insecurity reflected limited or uncertain availability of safe water and/or nutritious food or lacking the ability to acquire safe water and/or nutritious food in socially acceptable ways [11, 22–24]. We used this model to calculate stabilized IPT weights using methods described elsewhere [25].

Estimates of syphilis seroprevalence in Nyakabare parish as measured by a positive TPHA test were generated by incorporating the IPT weights using the *svy* command in Stata (StataCorp LLC, College Station, Texas, USA). We assessed sensitivity to extreme IPT weights by excluding those individuals with weights at the 99th/1st, 95th/5th, and 90th/10th percentiles (Supplementary Table 1) [26]. We used univariable logistic regression to estimate the association between syphilis seroprevalence and pertinent covariates stratified by sex given the different ways that men and women consider and manage STIs. Covariates with α = .10 in the univariable logistic regression models were included in the multivariable logistic regression models, also stratified by sex. Statistical significance was defined as covariates with α = .05. Participants with missing data for any model covariates were excluded from analyses. Data were analyzed using Stata version 15 software.

### Ethics

All participants provided written informed consent to participate in study procedures. Ethical approval was obtained from the Mass General Brigham and Mbarara University of Science and Technology institutional review boards. Additional clearance was also obtained from the Uganda National Council of Science and Technology and the President's Office.

## RESULTS

**Figure 1.**
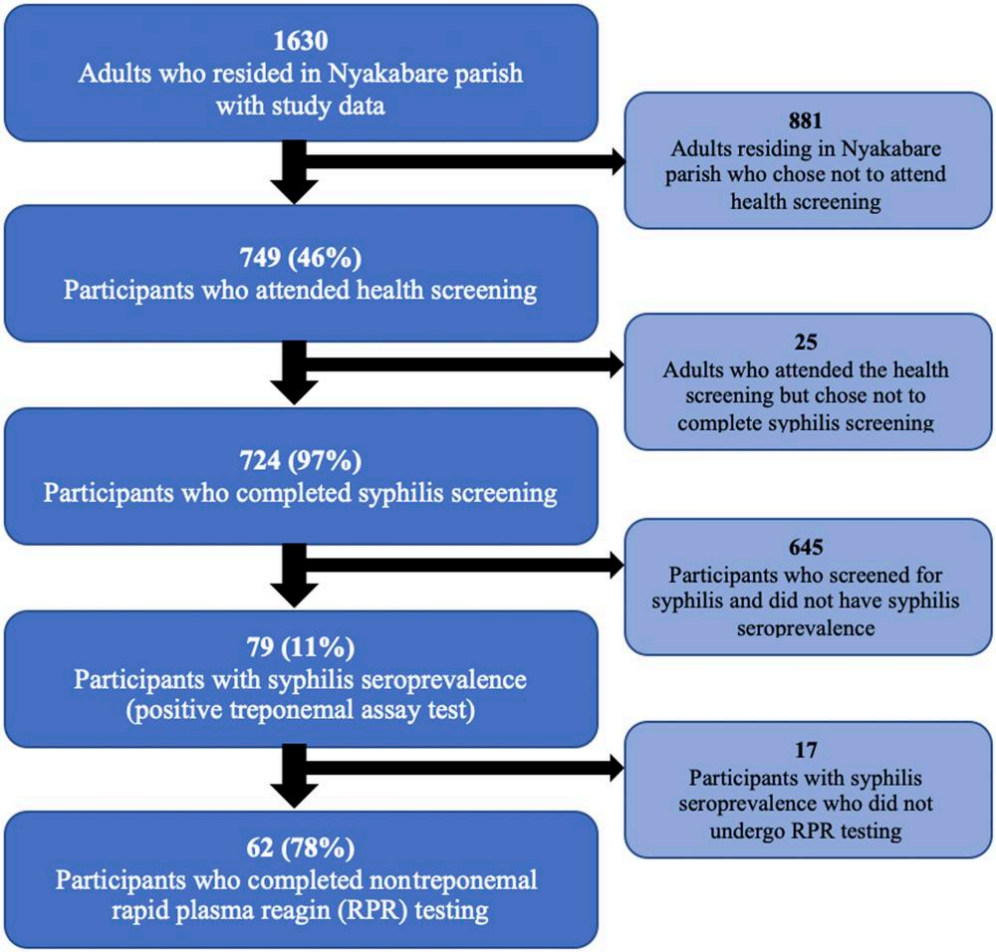
Flow diagram of participant selection demonstrating eligible participants, proportion of eligible participants who attended a health screening fair, proportion of participants who completed syphilis screening, proportion of participants with syphilis seroprevalence, and proportion of participants who completed rapid plasma reagin (RPR) testing.

All Nyakabare parish adult residents were invited to participate in the 2019 health screening fairs. Of the 1630 enumerated residents participating in the cohort study in 2019, 749 of 1630 (45.9%) attended the 2019 health screening. Of these attendees, 724 (96.7%) participated in the syphilis screening (Figure 1). Compared to the population of residents who did not participate in the health screenings, the population of attendees were of older age, less likely to be circumcised, more likely to be women, more likely to be married/cohabitating, less educated, less wealthy, had worse self-reported health, and had more experiences of intimate partner violence (Supplementary Table 2). Among attendees, those who opted into syphilis screening were more likely to have formal education than those who opted out of syphilis screening (Supplementary Table 3).

Based on the IPT-weighted model, the population prevalence of HIV was 11.4%, 17.8% had STI symptoms at the time of screening, nearly one-third (32.4%) reported a prior lifetime STI, 10.5% reported 2 or more sexual partners in the prior month, and 26.3% of men were circumcised (Table 1).

**Table 1.** Characteristics of Participants Who Participated in a Population-Based Syphilis Screening in a Rural Community in Southwestern Uganda

Characteristic	Total Participants, Number	Number	Unweighted Population Estimates	Weighted Population Estimates
Age, y, mean ± SD	737	…	42.9 ± 15.6	40.6 ± 15.5
Sex	724			
Female	…	456	63.0%	55.5%
Male	…	268	37.0%	44.5%
Relationship status	723			
Married or cohabitating	…	502	69.4%	63.9%
Separated or divorced	…	143	19.8%	17.3%
Single, never married	…	78	10.8%	18.8%
Pregnancy status	450			
Not currently pregnant	…	431	95.8%	95.3%
Currently pregnant	…	19	4.2%	4.7%
Education	724			
None	…	100	13.8%	13.3%
Primary	…	439	60.5%	53.1%
Secondary school and higher	…	186	25.7%	33.5%
Household asset wealth	724			
Poorest	…	162	22.4%	20.1%
Poorer	…	160	22.1%	20.3%
Middle	…	144	19.9%	19.7%
Less poor	…	142	19.6%	20.0%
Least poor	…	116	16.0%	20.0%
Self-reported overall health	723			
Very bad	…	2	0.3%	1.0%
Bad	…	158	21.9%	18.6%
Good	…	468	64.7%	64.6%
Very good	…	95	13.1%	15.9%
Circumcised (men only)	231			
Not circumcised	…	182	78.8%	73.7%
Circumcised	…	49	21.2%	26.3%
HIV serostatus	700			
HIV negative	…	621	88.7%	88.6%
HIV positive	…	79	11.3%	11.4%
New HIV diagnosis	626			
No new HIV diagnosis	…	616	98.4%	98.8%
New HIV diagnosis	…	10	1.6%	1.2%
Current STI symptoms	…			
No STI symptoms	724	429	59.3%	82.2%
STI symptoms	…	295	40.8%	17.8%
Lifetime STI experience				
No prior lifetime STI	710	489	68.9%	67.6%
Prior lifetime STI	…	221	31.1%	32.4%
No. of sexual partners in the past 1 month	501			
0	…	27	5.4%	5.8%
1	…	435	86.8%	83.7%
≥2	…	39	7.8%	10.5%
Condom use at last sex	594			
No condom use	…	541	91.1%	90.8%
Condom use	…	53	8.9%	9.2%
Transactional sex in the past 12 mo	692			
No transactional sex	…	619	89.5%	87.6%
Transactional sex	…	73	10.6%	12.4%
Threats of or experienced IPV in the past 3 mo	212			
Did not experience IPV	…	126	59.4%	64.8%
Experienced IPV	…	86	40.6%	35.2%
Last drank alcohol	722			
Never	…	336	46.5%	46.1%
Within the past 12 mo	…	210	29.1%	29.8%
>12 mo	…	176	24.4%	24.1%

Abbreviations: HIV, human immunodeficiency virus; IPV, intimate partner violence; SD, standard deviation; STI, sexually transmitted infection.

The IPT-weighted syphilis population seroprevalence was 10.6% (95% confidence interval [CI]: 8.4%–13.4%) (Table 2). Of the 79 participants with syphilis seroprevalence, 62 completed RPR testing: 35.9% (95% CI, 23.7%–50.3%) had a nonreactive titer, 53.8% (95% CI, 39.9%–67.1%) had a 1:1 titer, 9.2% (95% CI, 4.2%–18.7%) had a 1:2 titer, 1.1% (95% CI, .1%–8.1%) had a 1:4 titer, and no participants had a ≥1:8 concentrated titer. Seventeen participants did not undergo RPR testing due to administrative error. Additionally, among participants with a positive TPHA, 33 of 79 (41.8%) reported current STI symptoms and 27 of 77 (35%) reported prior lifetime STI.

**Table 2.** *Treponema pallidum* Hemagglutination Assay Positivity (Syphilis Seroprevalence) and Rapid Plasma Reagin Prevalence

Test Type	Number/Total Number	Unweighted Prevalence	Weighted Prevalence Estimates Without Trimming
		% (95% CI)	% (95% CI)
TPHA^a^			
Negative	644/723	89.1 (86.6–91.3)	89.4 (86.6–91.6)
Positive	79/723	10.9 (8.7–13.4)	10.6 (8.4–13.4)
RPR^b^			
Nonreactive	22/62	35.5 (23.7–48.7)	35.9 (23.7–50.3)
1:1 titer	32/62	51.6 (38.6–64.5)	53.8 (39.9–67.1)
1:2 titer	7/62	11.3 (4.7–21.9)	9.2 (4.2–18.7)
1:4 titer	1/62	1.6 (.0–8.7)	1.1 (.1–8.1)

Abbreviations: CI, confidence interval; RPR, rapid plasma reagin; TPHA, *Treponema pallidum* hemagglutination assay.

^a^One participant had an inconclusive TPHA.

^b^Sixty-two participants completed RPR testing and had data available for weighting.

Based on the IPT-weighted model, 19.4% (95% CI, 5.7%–49.2%) of pregnant people (reported during the health screening) had syphilis seroprevalence compared to 10.2% (95% CI, 7.6%–13.6%) of nonpregnant people (Table 3). More uncircumcised men (10.8% [95% CI, 6.9%–16.4%]) had syphilis seroprevalence compared to circumcised men (6.8% [95% CI, 1.6%–24.7%]). More participants with no formal education (14.9% [95% CI, 8.8%–24.1%]) had syphilis seroprevalence compared to people with primary (11.3% [95% CI, 8.4%–15.1%]) or secondary (7.9% [95% CI, 4.6%–13.2%]) education. Additionally, 19.7% (95% CI, 11.7%–31.3%) of people known to be living with HIV and 15.7% (95% CI, 2.0%–62.9%) of people with a new HIV diagnosis through the health screening had syphilis seroprevalence compared to 9.5% (95% CI, 7.3%–12.2%) of people without HIV.

**Table 3.** *Treponema pallidum* Hemagglutination Assay Positivity Prevalence (Syphilis Seroprevalence) Among Different Participant Categories

Characteristic	Number/Total Number	Unweighted Prevalence, % (95% CI)	Weighted Prevalence Estimates, % (95% CI)
Sex			
Female	49/456	10.7 (8.1–14.0)	10.5 (7.9–13.9)
Male	30/267	11.2 (7.7–15.7)	10.8 (7.3–15.7)
Relationship status			
Married or cohabitating	47/502	9.4 (7.0–12.3)	8.7 (6.4–11.6)
Separated or divorced	23/143	16.1 (10.5–23.1)	16.4 (10.7–24.3)
Single, never married	9/77	11.7 (5.5–21.0)	11.9 (6.0–22.5)
Pregnancy status			
Not currently pregnant	46/431	10.7 (7.9–14.0)	10.2 (7.6–13.6)
Currently pregnant	3/19	15.8 (3.4–39.6)	19.4 (5.7–49.2)
Education			
None	14/100	14.0 (7.9–22.4)	14.9 (8.8–24.1)
Primary	48/437	11.0 (8.2–14.3)	11.3 (8.4–15.1)
Secondary school or higher	17/186	9.1 (5.4–14.2)	7.9 (4.6–13.2)
Household asset wealth			
Poorest	22/162	13.6 (8.7–19.8)	13.8 (8.7–21.0)
Poorer	14/160	8.8 (4.9–14.2)	8.6 (5.0–14.2)
Middle	15/144	10.4 (5.9–16.6)	10.0 (5.9–16.4)
Less poor	19/142	13.4 (8.3–20.1)	13.4 (8.2–21.1)
Least poor	9/115	7.8 (3.6–14.3)	7.5 (3.6–15.2)
Self-reported overall health			
Very bad	0/2	0 (.0–84.2)	0
Bad	16/158	10.1 (5.9–15.9)	9.2 (5.4–15.3)
Good	56/468	12.0 (9.2–15.3)	12.1 (9.3–15.7)
Very good	7/94	7.4 (3.0–14.7)	7.9 (3.6–16.4)
Circumcised (men only)			
Not circumcised	22/182	12.1 (7.7–17.7)	10.8 (6.9–16.4)
Circumcised	2/49	4.1 (.5–14.0)	6.8 (1.6–24.7)
HIV serostatus			
HIV negative	62/620	10.0 (7.8–12.6)	9.5 (7.3–12.2)
HIV positive	14/79	17.7 (10.0–27.9)	19.7 (11.7–31.3)
New HIV diagnosis			
No new HIV diagnosis	64/615	10.4 (8.1–13.1)	10.3 (8.0–13.3)
New HIV diagnosis	3/10	30.0 (6.7–65.2)	15.7 (2.0–62.9)
Current STI symptoms			
No STI symptoms	46/428	10.7 (8.0–14.1)	10.4 (7.6–14.1)
STI symptoms	33/295	11.2 (7.8–15.4)	11.2 (7.9–15.8)
Lifetime STI experience			
No prior lifetime STI	50/488	10.2 (7.7–13.3)	9.4 (7.1–12.5)
Prior lifetime STI	27/221	12.2 (8.2–17.3)	12.9 (8.5–19.0)
Prior STI treatment			
Never treated	0/11	0 (.0–28.5)	0
Sometimes treated	2/23	8.7 (1.1–28.0)	11.0 (2.5–37.7)
Always treated	24/187	12.8 (8.4–18.5)	12.8 (8.2–19.4)
Partner STI treatment			
None treated	8/57	14.0 (6.3–25.8)	14.5 (6.8–28.0)
Some treated	1/20	5.0 (.1–24.9)	5.0 (.6–33.2)
All treated	13/119	10.9 (5.9–18.0)	10.8 (5.8–19.5)
No. of sexual partners in the past 1 month			
0	2/27	7.4 (.9–24.3)	6.4 (1.4–24.2)
1	41/435	9.4 (6.8–12.6)	8.7 (6.3–11.9)
≥2	4/39	10.3 (2.9–24.2)	9.3 (3.2–24.2)
Condom use at last sex			
No condom use	52/540	9.6 (7.3–12.4)	9.6 (7.2–12.7)
Condom use	7/53	13.2 (5.5–25.3)	11.9 (5.0–25.7)
Transactional sex in the past 12 mo			
No transactional sex	69/619	11.1 (8.8–13.9)	10.8 (8.4–13.7)
Transactional sex	5/72	6.9 (2.3–15.5)	7.0 (2.7–17.0)
Threats of or experienced IPV in the past 3 mo			
Did not experience IPV	17/126	13.5 (8.1–20.7)	12.7 (7.4–20.9)
Experienced IPV	8/85	9.4 (4.2–17.7)	9.3 (4.1–19.8)
Last drank alcohol			
Never	38/336	11.3 (8.1–15.2)	11.6 (8.4–16.0)
Last 12 mo	24/210	11.4 (7.5–16.5)	11.0 (6.9–16.9)
>12 mo	17/175	9.7 (5.8–15.1)	8.3 (5.1–13.4)

Abbreviations: CI, confidence interval; HIV, human immunodeficiency virus; IPV, intimate partner violence; STI, sexually transmitted infection.

In the IPT-weighted multivariable regression models among women, syphilis seroprevalence was lower among those with primary (adjusted odds ratio [aOR], 0.38 [95% CI, .23–.63]) and secondary education (aOR, 0.44 [95% CI, .20–.94]) compared to no education. Syphilis seroprevalence was associated with HIV seropositivity (aOR, 4.00 [95% CI, 1.58–10.13]) and having ≥2 sexual partners in the prior month (aOR, 7.49 [95% CI, 1.18–47.67]) (Table 4). Among men, syphilis seroprevalence was associated with HIV seropositivity (aOR, 2.85 [95% CI, 1.11–7.29]) (Table 5). Syphilis seroprevalence was also associated with being separated or divorced (aOR, 2.81 [95% CI, .93–8.50]) and with having STI symptoms (aOR, 1.86 [95% CI, .93–3.71]), but these coefficients were imprecisely estimated.

**Table 4.** Inverse Probability of Treatment-Weighted Unadjusted and Adjusted *Treponema pallidum* Hemagglutination Assay Correlates in Multivariable Logistic Regression Among Women Participating in a Population-Based Syphilis Screening in a Rural Community in Southwestern Uganda

Characteristic	Number	Unadjusted			Adjusted		
		Crude OR	(95% CI)	*P* Value	Adjusted OR	(95% CI)	*P* Value
Age (per y)	432	1.03	(1.01–1.05)	<.01	1.01	(.97–1.05)	.70
Relationship status	432			.21			
Married or cohabitating		Ref	…		…	…	
Separated or divorced		1.41	(.95–2.10)		…	…	
Single, never married		0.82	(.18–3.63)		…	…	
Education^a^	432			<.01			
None		Ref	…		Ref	…	
Primary		0.60	(.33–1.08)		0.38	(.23–.63)	<.01
Secondary school or higher		0.37	(.20–.68)		0.44	(.20–.94)	.03
Household asset wealth	432			.11			
Poorest		Ref	…		…	…	
Poorer		0.98	(.33–2.91)		…	…	
Middle		0.95	(.49–1.86)		…	…	
Less poor		1.30	(.39–4.34)		…	…	
Least poor		0.42	(.10–1.81)		…	…	
HIV serostatus	432			.02			<.01
HIV positive		Ref	…		Ref	…	
HIV negative		1.94	(1.11–3.39)		4.00	(1.58–10.13)	
Current STI symptoms	432			.36			
No STI symptoms		Ref	…		…	…	
STI symptoms		0.84	(.57–1.23)		…	…	
Lifetime STI experience	423			.21			
No prior lifetime STI		Ref	…		…	…	
Prior lifetime STI		1.39	(.83–2.32)		…	…	
No. of sexual partners in the past 1 month	295			<.01			
0		Ref	…		Ref	…	
1		0.97	(.27–3.53)		1.08	(.29–3.98)	.91
≥2		7.23	(1.08–48.27)		7.49	(1.18–47.67)	.03
Condom use at last sex	338			.51			
No condom use		Ref	…		…	…	
Condom use		1.46	(.48–4.45)		…	…	
Transactional sex in the past 12 mo	411			.30			
No transactional sex		Ref	…		…	…	
Transactional sex		0.36	(.05–2.51)		…	…	

Abbreviations: CI, confidence interval; HIV, human immunodeficiency virus; OR, odds ratio; STI, sexually transmitted infection.

**Table 5.** Inverse Probability of Treatment–Weighted Unadjusted and Adjusted *Treponema pallidum* Hemagglutination Assay Correlates in Multivariable Logistic Regression Among Men Participating in a Population-Based Syphilis Screening in a Rural Community in Southwestern Uganda

Characteristic	Number	Unadjusted			Adjusted		
		Crude OR	(95% CI)	*P* Value	Adjusted OR	(95% CI)	*P* Value
Age (per year)	252	1.02	(.99–1.05)	.24	…	…	…
Relationship status	252			.02			
Married or cohabitating		Ref	…		…	…	
Separated or divorced		4.48	(1.54–13.0)		2.81	(.93–8.50)	.07
Single, never married		2.05	(.44–9.59)		2.41	(.56–10.43)	.24
Education	252			.59			
None		Ref	…		…	…	
Primary school		1.23	(.32–4.67)		…	…	
Secondary school or higher		0.85	(.27–2.66)		…	…	
Household asset wealth	252			<.01			
Poorest		Ref	…		Ref	…	
Poorer		0.25	(.06–1.01)		0.41	(.10–1.76)	.23
Middle		0.45	(.22–.92)		0.66	(.27–1.61)	.37
Less poor		0.68	(.28–1.66)		0.84	(.31–2.25)	.73
Least poor		0.47	(.12–0.41)		0.62	(.22–1.72)	.36
Circumcised	218			.43			
Not circumcised		Ref	…		…	…	
Circumcised		0.61	(.17–2.10)		…	…	
HIV serostatus	252			.08			
HIV negative		Ref	…		Ref	…	
HIV positive		3.13	(.86–11.40)		2.85	(1.11–7.29)	.03
Current STI symptoms	252			.02			
No STI symptoms		Ref	…		Ref	…	
STI symptoms		1.84	(1.09–3.11)		1.86	(.93–3.71)	.08
Lifetime STI experience	248			.50			
No prior lifetime STI		Ref	…		…	…	
Prior lifetime STI		1.47	(.49–4.42)		…	…	
No. of sexual partners in the past 1 mo	181			.54			
0		…	…		…	…	
1		Ref	…		…	…	
≥2^a^		1.58	(.36–6.87)		…	…	
Condom use at last sex	226			.87			
No condom use		Ref	…		…	…	
Condom use		1.11	(.32–3.85)		…	…	
Transactional sex in the past 12 mo	243			.12			
No transactional sex		Ref	…		…	…	
Transactional sex		0.76	(.54–1.08)		…	…	

Abbreviations: CI, confidence interval; HIV, human immunodeficiency virus; OR, odds ratio; STI, sexually transmitted infection.

^a^Two or more sexual partners in the past 1 month omitted due to collinearity with 1 sexual partner in the past 1 month.

## DISCUSSION

In this cross-sectional, community-based study conducted in rural Uganda, we estimated a syphilis population seroprevalence of 10.6%, indicating high lifetime syphilis exposure in this population. In a setting where RPR is not routinely performed, we found that participants with syphilis seroprevalence had negative or low-titer RPR results. To contextualize these findings, the background weighted HIV prevalence of 11.3% in our setting was higher than the estimated Uganda national prevalence of 5.1%, potentially demonstrating the known synergy between these 2 pathogens [27]. Although the syphilis prevalence in the general community was high (10.6%), the weighted syphilis prevalence rates among people with HIV (PWH) (19.7%) and pregnant people (19.4%) were striking. While PWH and pregnant people should continue to be priority populations for screening, our findings highlight the importance of also integrating syphilis screening into novel outreach efforts focused on the general community.

To our knowledge, a syphilis seroprevalence >10% has not been described in a general population sample. Typically, the syphilis prevalence is higher among populations with a high HIV prevalence, including men who have sex with men, people participating in transactional sex, and—in Uganda specifically—people in fishing communities. A recent study near Rakai, Uganda, documented the syphilis prevalence using treponemal testing followed by nontreponemal testing in a population-based census. They found a syphilis seroprevalence of 7% and an HIV prevalence of 14% among 919 participants in a non-fishing community, and a syphilis seroprevalence of 24% and an HIV prevalence of 40% among 906 participants in a fishing community [28]. A recent meta-analysis found the syphilis prevalence across 53 studies with 211 976 PWH in sub-Saharan Africa to be 7.3% (95% CI, 6.3%–8.5%), with the East Africa region having the highest prevalence at 10.5% (95% CI, 8.0%–13.1%) [29]. Additionally, the African Cohort Study (AFRICOS) cohort assessed nontreponemal testing followed by treponemal testing (the traditional algorithm) among 2818 PWH in Uganda, Kenya, Tanzania, and Nigeria. They found a 5.3% prevalence of participants with positive nontreponemal testing (an RPR titer of ≥1:2) and 3.1% of participants with both positive nontreponemal and treponemal testing [30]. Comparisons among syphilis studies are difficult given the variation in testing used (eg, a single test vs combination of treponemal and nontreponemal testing), testing sequence, and definition of a positive test. However, our syphilis prevalence in the general community equals the syphilis prevalence of PWH elsewhere.

Novel methods are needed to enhance syphilis screening. One strategy is to integrate syphilis screening into existing HIV screening programs including community screening events, HIV prevention/preexposure prophylaxis programs, and family planning programs for nonpregnant people [31, 32]. Several, available dual POC HIV/syphilis tests on the market could be utilized [33]. Additionally, strengthening primary care infrastructure in resource-limited settings [34, 35] would allow broader populations to access preventive care inclusive of syphilis screening. Finally, recent work has demonstrated the efficacy of doxycycline postexposure prophylaxis in reducing syphilis incidence among men who have sex with men and transgender women; more research is needed to evaluate the efficacy of doxycycline in other populations [36, 37].

Reasons for high syphilis prevalence in Uganda and East Africa are unclear, though the limitations of POC treponemal antibody testing and misconceptions regarding syphilis transmission and treatment likely contribute. Our qualitative research in this setting observed that participants in HIV-serodiscordant relationships were confused regarding syphilis transmission [38–40]. Participants often believed that curable STIs were spread through nonsexual methods including sharing toilets and clothing. Further, the idea of perinatal transmission has been conflated with the idea of genetic transmission across Uganda, with many of our participants stating that syphilis is a genetic disease and therefore incurable. These beliefs are likely reinforced by the limitations of syphilis testing with POC treponemal antibodies that remain positive for decades despite adequate treatment, limiting the ability to demonstrate syphilis cure. Such beliefs regarding the incurable nature of syphilis could dissuade people with a syphilis diagnosis from seeking appropriate treatment. Additionally, stockouts of POC treponemal tests and benzathine penicillin likely contribute to rising syphilis infections in Uganda and worldwide [41].

Even when people with a syphilis diagnosis access appropriate diagnostics and treatment, resources are often not robust for additional prevention efforts. A study based in Kampala antenatal clinics assessing the effectiveness of syphilis partner notification strategies found that among 442 pregnant women with syphilis, only 18% of male partners attended antenatal clinics and received syphilis treatment [42]. Another potential reason for the high syphilis prevalence in our setting could be related to low rates of voluntary male medical circumcision. A study assessing the effect of voluntary male medical circumcision on syphilis among 42 109 men aged 15–59 years in Uganda, Tanzania, Zimbabwe, and Zambia found that medically circumcised men had a lower odds of a positive treponemal test compared to men who were not circumcised in the univariable model only. The prevalence of men with any type of circumcision in Uganda has ranged from 22% to 27% [43, 44] (similar to our weighted prevalence of 26%). In our study setting, comprehensive public education campaigns on syphilis transmission and the benefits of voluntary male medical circumcision could potentially support syphilis treatment and prevention. However, this information could also result in increased stigma with people vulnerable to syphilis avoiding care. Ultimately, an accurate, accessible, combination nontreponemal and treponemal POC test is needed to prevent the confusion of continued positive treponemal tests despite treatment. Regardless of the setting, understanding people's beliefs and cultural practices related to sexual and reproductive health inclusive of STIs is a crucial step to designing an effective STI care program.

We found that participants with syphilis seroprevalence had either nonreactive or low RPR titers. Few studies assessing syphilis seroprevalence have assessed RPR, likely due to the increased laboratory capacity and skill needed to perform this test. RPR testing is dependent on laboratory technician experience and expertise with results varying with temperature and humidity [45]; thus, RPR results can vary among settings, and these factors may have contributed to our low RPR titers. RPR is most useful when comparing results for an individual over time to allow clinicians to assess an adequate syphilis treatment response (a 4-fold decline in titer; eg, a decline from 1:16 to 1:4). Positive treponemal tests with low titer or negative nontreponemal testing are possible in those previously treated for syphilis, those with very early syphilis, and those with late syphilis. As most participants with positive TPHA in our study reported no current STI symptoms, early syphilis was unlikely for most participants. Additionally, most participants reported no prior STI, decreasing the likelihood of previously treated, serofast syphilis (continued nontreponemal reactivity despite treatment). Because the median age of our participants was 40.7 years (standard deviation of 15.6 years, meaning many participants were outside the age in which they would attend antenatal clinics) and only a minority were living with HIV (thus without access to syphilis screening), it is likely that many of our participants had late latent syphilis as RPR titers naturally wane over time even without treatment [46]. The study evaluating syphilis prevalence in Rakai, Uganda, found that 80% and 61% of those with confirmed syphilis seroprevalence in the non-fishing and fishing communities, respectively, had low-titer (<1:8) syphilis [28], likely indicating high rates of active syphilis compared to the communities in this study.

Additionally, our other findings from the multivariable regression analysis support known associations of syphilis prevalence in the literature. Specifically, we found that living with HIV, having ≥2 sexual partners, STI symptoms, less education, and being separated/divorced were significantly associated with syphilis prevalence [5, 30, 47–49].

Our study has several limitations. This study assessed syphilis prevalence in a single Ugandan parish, thus limiting the generalizability of our findings. The primary outcome of our study was syphilis seroprevalence as measured by a positive TPHA. While we measured nontreponemal antibodies via RPR, given its known measurement variability, RPR was not our primary outcome. Focusing on 1 antibody test versus testing for both treponemal and nontreponemal antibodies is known to result in a 2-fold higher odds of test positivity as described by Smolak et al in a global meta-analysis of syphilis [50]. This approach could partially explain our high syphilis prevalence in comparison with studies defining syphilis as having both a positive treponemal and nontreponemal test. Our use of the reverse algorithm with treponemal TPHA followed by nontreponemal RPR testing generally facilitates comparison among studies (as many studies only perform treponemal testing given the extra laboratory capacity needed to perform RPR), though varied testing options and algorithms complicate comparison. Our study was cross-sectional, and thus we were unable to follow participants to assess late latent syphilis treatment completion, repeat RPR titers, or partner evaluation and treatment. Further complicating our analysis is the potential for serologic cross-reactivity with *T pallidum* subspecies *pertenue*, the bacterium leading to yaws. However, yaws underwent mass eradication efforts in the mid-20th century and is not currently endemic in Uganda [51]. Finally, cultural and legal constraints prevented questions regarding sexual orientation, limiting our ability to describe syphilis prevalence among sexual and gender minorities.

## CONCLUSIONS

Our findings demonstrate a high population prevalence of syphilis in Nyakabare parish in rural, southwestern Uganda, highlighting the urgent need for community-wide screening. However, many resource-limited settings lack the infrastructure and resources to implement routine STI screening. Systems instead focus on populations with existing clinic infrastructure who are most vulnerable to syphilis comorbidities, including PWH and pregnant people. Indeed, our syphilis prevalence was even higher among PWH and pregnant people compared to the general community. Settings with a high population prevalence of syphilis require Ministry of Health support to concurrently invest syphilis screening capacity in existing HIV and antenatal clinics as well as primary care infrastructure to allow preventive healthcare inclusive of generalized syphilis screening and treatment. Further, access to an accurate and affordable POC, dual treponemal and nontreponemal antibody test will allow the effective monitoring of syphilis treatment outcomes.

## Supplementary Material

ofaf290_Supplementary_Data

## Supplementary Data

Supplementary materials are available at *Open Forum Infectious Diseases* online. Consisting of data provided by the authors to benefit the reader, the posted materials are not copyedited and are the sole responsibility of the authors, so questions or comments should be addressed to the corresponding author.

## Notes

***Acknowledgments.*** We thank Dr Edward Hook (Division of Infectious Diseases, Heersink School of Medicine, University of Alabama at Birmingham) and Dr Kathy Hsu (Division of Pediatric Infectious Diseases, Boston Medical Center; and the Division of STD Prevention and HIV/AIDS Surveillance, Massachusetts Department of Public Health) for their expert guidance in the laboratory diagnostics. In addition to the named study authors, we thank additional members of the HopeNet study team for their contributions to data collection and study administration: Elizabeth Namara, Viola Kyokunda, Allen Kiconco, Immaculate Ninsiima, Patrick Lukwago, Juliet Mercy, and Phiona Ahereza. We also thank the participants for their contribution to this study.

***Author contributions.*** A. C. T. and B. K. conceptualized the parent study. P. C. conceptualized this analysis of data from the parent study. C. B., J. M. R., and E. N. S. participated in data management and data curation. C. B. oversaw the data collection and project administration. J. K., P. A., and P. G. collected data. G. M. oversaw laboratory operations. N. M. and P. C. performed data analysis. A. C. T. provided supervision on the analytic approach. P. C. wrote the initial draft of the manuscript. All authors contributed to manuscript writing and editing.

***Data availability.*** The data are not publicly available.

***Financial support.*** The study was funded by the US National Institutes of Health (NIH) (grant number R01MH113494-01) and Friends of a Healthy Uganda (to A.C. T.). P. C. acknowledges salary support from the NIH (grant numbers T32AI007433, D43TW010543, and K23MH126771-01A1, and P30AI060354 to the Harvard University Center for AIDS Research) and a Harvard Global Health Institute, Burke Global Health Fellowship. A. C. T. acknowledges salary support from the NIH (award number K24DA061696-01).

***Potential conflicts of interest.*** A. C. T. reports receiving an honorarium from Elsevier for his work as Co–Editor in Chief of the Elsevier-owned journal SSM–Mental Health. All other authors report no potential conflicts of interest.
