# Supplementary material for: Population Prevalence and Correlates of Syphilis in Rural, Southwestern Uganda
Source: Open Forum Infect Dis. 2025 May 28;12(5):ofaf290. doi: 10.1093/ofid/ofaf290 (PMC12117536; doi:10.1093/ofid/ofaf290)
Supplement: ofaf290_Supplementary_Data [file ofaf290_supplementary_data.docx]

**Supplementary Table 1.** Treponema pallidum hemagglutination assay (TPHA) positivity prevalence (syphilis seroprevalence) among different participant categories with sensitivity to extreme IPTW weights by trimming those with weights at the 99th/1st, 95th/5th and 90th/10th percentiles.

| Characteristic | N/total | Unweighted prevalence  % (95% CI) | Weighted prevalence  estimates without trimming  % (95% CI) | Weighted prevalence  estimates with trimming % (95% CI) | | |
| --- | --- | --- | --- | --- | --- | --- |
|  |  |  |  | 99^th^, 1^st^ percentile | 95^th^, 5^th^ percentile | 90^th^, 10^th^ percentile |
| TPHA*  Negative  Positive | 644/723  79/723 | 89.1% (86.6-91.3)  10.9% (8.7-13.4) | 89.4% (86.6-91.6)  10.6% (8.4-13.4) | 89.3% (86.5-91.5)  10.7% (8.5-13.5) | 89.1% (86.4-91.4)  10.9% (8.6-13.6) | 89.1% (86.4-91.3)  10.9% (8.7-13.6) |
| RPR**  Nonreactive  1:1 titer  1:2 titer  1:4 titer | 22/62  32/62  7/62  1/62 | 35.5% (23.7-48.7)  51.6% (38.6-64.5)  11.3% (4.7-21.9)  1.6% (0.0-8.7) | 35.9% (23.7-50.3)  53.8% (39.9-67.1)  9.2% (4.2-18.7)  1.1% (0.1-8.1) | 36.0% (23.7-50.3)  53.8% (39.8-67.1)  9.2% (4.2-18.7)  1.1% (0.1-8.1) | 36.3% (24.2-50.5)  53.2% (39.5-66.4)  9.3% (4.3-19.0)  1.1% (0.2-8.2) | 36.4% (24.4-50.3)  52.9% (39.4-66.0)  9.6% (4.5-19.3)  1.2% (0.2-8.3) |
| Age in years (mean, standard deviation) | - | 50.1 +/- 16.9 (46.3-53.9) | 46.6 +/- 17.2  (42.1-51.1) | 46.6 +/- 17.2  (42.1-51.1) | 47.1 +/- 17.1 (42.8-51.4) | 47.5 +/- 17.0  (43.4-51.7) |
| Sex (%)  Women  Men | 49/456  30/267 | 10.7% (8.1-14.0)  11.2% (7.7-15.7) | 10.5% (7.9-13.9)  10.8% (7.3-15.7) | 10.5% (7.9-13.9)  11.0% (7.5-16.0) | 10.6% (8.0-14.0)  11.2% (7.7-16.1) | 10.7% (8.6-14.1)  11.1% (7.7-15.9) |
| Relationship status (%)  Married or cohabitating  Separated or divorced  Single, never married | 47/502  23/143  9/77 | 9.4% (7.0-12.3)  16.1% (10.5-23.1)  11.7% (5.5-21.0) | 8.7% (6.4-11.6)  16.4% (10.7-24.3)  11.9% (6.0-22.5) | 8.7% (6.4-11.6)  16.4% (10.7-24.3)  11.9% (6.0-22.5) | 8.7% (6.5-11.6)  16.6% (10.9-24.5)  13.3% (6.8-24.3) | 8.8% (6.5-11.7)  16.6% (10.9-24.5)  13.3% (6.9-24.1) |
| Pregnancy status (%)  Not currently pregnant  Currently pregnant | 46/431  3/19 | 10.7% (7.9-14.0)  15.8% (3.4-39.6) | 10.2% (7.6-13.6)  19.4% (5.7-49.2) | 10.2% (7.6-13.6)  19.4% (5.6-49.1) | 10.3% (7.7-13.7)  19.3% (5.6-48.9) | 10.4% (7.7-13.8)  19.2% (5.6-48.9) |
| Education (%)  None  Primary  ≥Secondary school | 14/100  48/437  17/186 | 14.0% (7.9-22.4)  11.0% (8.2-14.3)  9.1% (5.4-14.2) | 14.9% (8.8-24.1)  11.3% (8.4-15.1)  7.9% (4.6-13.2) | 15.0% (8.9-24.2)  11.4% (8.5-15.2)  8.0% 4.7-13.4) | 15.1% (9.0-24.4)  11.4% (8.5-15.1)  8.4% (4.9-13.8) | 15.3% (9.1-24.5)  11.4% (8.5-15.0)  8.3% (4.9-13.7) |
| Household asset wealth (%)  Poorest  Poorer  Middle  Less poor  Least poor | 22/162  14/160  15/144  19/142  9/115 | 13.6% (8.7-19.8)  8.8% (4.9-14.2)  10.4% (5.9-16.6)  13.4% (8.3-20.1)  7.8% (3.6-14.3) | 13.8% (8.7-21.0)  8.6% (5.0-14.2)  10.0% (5.9-16.4)  13.4% (8.2-21.1)  7.5% (3.6-15.2) | 13.8% (8.7-21.1)  8.6% (5.0-14.2)  10.0% (5.9-16.4)  13.5% (8.3-21.2)  7.9% (3.7-15.8) | 13.9% (8.9-21.2)  8.6% (5.0-14.2)  10.2% (6.1-16.6)  13.7% (8.5-21.4)  7.9% (3.8-15.5) | 13.7% (8.8-20.7)  8.6% (5.1-14.3)  10.4% (6.2-16.9)  13.7% (8.6-21.3)  7.8% (3.9-15.3) |
| Self-reported overall health (%)  Very bad  Bad  Good  Very good | 0/2  16/158  56/468  7/94 | 0% (0.0-84.2)  10.1% (5.9-15.9)  12.0% (9.2-15.3)  7.4% (3.0-14.7) | 0%  9.2% (5.4-15.3)  12.1% (9.3-15.7)  7.9% (3.6-16.4) | 0%  9.1% (5.3-15.4)  11.8% (9.0-15.3)  8.7% (3.7-18.8) | 0%  9.2% (5.3-15.5)  12.0% (9.2-15.6)  8.3% (3.7-17.5) | 0%  9.2% (5.4-15.3)  12.1% (9.3-15.7)  7.9% (3.6-16.4) |
| Circumcised (%) (men only)  Not circumcised  Circumcised | 22/182  2/49 | 12.1% (7.7-17.7)  4.1% (0.5-14.0) | 10.8% (6.9-16.4)  6.8% (1.6-24.7) | 10.9% (7.0-16.6)  7.1% (1.7-25.4) | 11.2% (7.2-17.0)  6.5% (1.6-23.3) | 11.3% (7.3-17.1)  5.9% (1.4-21.6) |
| HIV serostatus (%)  Not living with HIV  Living with HIV | 62/620  14/79 | 10.0% (7.8-12.6)  17.7% (10.0-27.9) | 9.5% (7.3-12.2)  19.7% (11.7-31.3) | 9.6% (7.4-12.4)  19.7% (11.7-31.3) | 9.7% (7.5-12.5)  19.8% (11.8-31.4) | 9.7% (7.5-12.4)  19.9% (11.9-31.5) |
| New HIV diagnosis (%)  No new HIV diagnosis  New HIV diagnosis | 64/615  3/10 | 10.4% (8.1-13.1)  30.0% (6.7-65.2) | 10.3% (8.0-13.3)  15.7% (2.0-62.9) | 10.4% (8.0-13.4)  17.6% (2.4-64.4) | 10.5% (8.2-13.5)  19.4% (2.9-66.2) | 10.5% (8.2-13.4)  20.2% (3.0-67.0) |
| Current STI symptoms (%)  No STI symptoms  STI symptoms | 46/428  33/295 | 10.7% (8.0-14.1)  11.2% (7.8-15.4) | 10.4% (7.6-14.1)  11.2% (7.9-15.8) | 10.3% (7.5-13.9)  11.2% (7.9-15.8) | 10.6% (7.8-14.3)  11.2% (7.9-15.7) | 10.6% (7.8-14.3)  11.2% (7.9-15.7) |
| Lifetime STI experience (%)  No prior lifetime STI  Prior lifetime STI | 50/488  27/221 | 10.2% (7.7-13.3)  12.2% (8.2-17.3) | 9.4% (7.1-12.5)  12.9% (8.5-19.0) | 9.5% (7.1-12.6)  13.0% (8.7-19.2) | 9.8% (7.3-12.9)  12.9% (8.6-18.8) | 9.9% (7.4-13.0)  12.6% (8.5-18.2) |
| Prior STI treatment (%)  Never treated  Yes sometimes treated  Yes always treated | 0/11  2/23  24/187 | 0% (0.0-28.5)  8.7% (1.1-28.0)  12.8% (8.4-18.5) | 0%  11.0% (2.5-37.7)  12.8% (8.2-19.4) | 0%  11.0% (2.5-37.7)  13.0% (8.3-19.6) | 0%  11.0% (2.5-37.7)  12.8% (8.3-19.2) | 0%  11.1% (2.5-37.7)  12.6% (8.3-18.8) |
| Partner STI treatment (%)  None treated  Some treated  All treated | 8/57  1/20  13/119 | 14.0% (6.3-25.8)  5.0% (0.1-24.9)  10.9% (5.9-18.0) | 14.5% (6.8-28.0)  5.0% (0.6-33.2)  10.8% (5.8-19.5) | 14.7% (7.0-28.4)  5.0% (0.6-33.2)  10.8% (5.8-19.5) | 15.1% (7.2-29.0)  5.0% (0.6-33.2)  10.4% (5.7-18.5) | 15.1% (7.3-28.8)  5.0% (0.6-33.2)  10.2% (5.6-18.0) |
| Number of sexual partners in the past 1 month (%)  0  1  ≥2 | 2/27  41/435  4/39 | 7.4% (0.9-24.3)  9.4% (6.8-12.6)  10.3% (2.9-24.2) | 6.4% (1.4-24.2)  8.7% (6.3-11.9)  9.3% (3.2-24.2) | 6.3% (1.4-24.3)  8.8% (6.4-12.0)  9.4% (3.2-24.3) | 6.3% (1.4-24.1)  8.8% (6.4-12.0)  9.6% (3.3-24.7) | 6.4% (1.4-24.3)  8.8% (6.4-12.0)  9.9% (3.5-25.2) |
| Condom use at last sex (%)  No condom use  Condom use | 52/540  7/53 | 9.6% (7.3-12.4)  13.2% (5.5-25.3) | 9.6% (7.2-12.7)  11.9% (5.0-25.7) | 9.6% (7.2-12.8)  12.2% (5.2-26.2) | 9.6% (7.2-12.7)  12.8% (5.5-27.1) | 9.6% (7.2-12.5)  12.8% (5.5-26.9) |
| Transactional sex in the past 12 months (%)  No transactional sex  Transactional sex | 69/619  5/72 | 11.1% (8.8-13.9)  6.9% (2.3-15.5) | 10.8% (8.4-13.7)  7.0% (2.7-17.0) | 10.8% (8.4-13.8)  7.2% (2.8-17.4) | 10.9% (8.5-13.8)  7.5% (2.9-17.9) | 10.9% (8.5-13.8)  7.4% (2.9-17.4) |
| Threats of or experienced intimate partner violence (IPV) in the past 3 months (%)  No experienced IPV  Experienced IPV | 17/126  8/85 | 13.5% (8.1-20.7)  9.4% (4.2-17.7) | 12.7% (7.4-20.9)  9.3% (4.1-19.8) | 13.1% (7.7-21.4)  9.3% (4.1-19.8) | 13.3% (8.0-21.4)  9.0% (4.2-18.8) | 13.3% (8.1-21.2)  8.6% (3.9-17.7) |
| Last drank alcohol (%)  Never  Last 12m  >12m | 38/336  24/210  17/175 | 11.3% (8.1-15.2)  11.4% (7.5-16.5)  9.7% (5.8-15.1) | 11.6% (8.4-16.0)  11.0% (6.9-16.9)  8.3% (5.1-13.4) | 11.8% (8.5-16.2)  11.0% (7.0-16.9)  8.4% (5.1-13.5) | 12.1% (8.7-16.5)  10.9% (7.0-16.7)  8.6% (5.2-13.7) | 12.1% (8.8-16.5)  10.8% (7.0-16.4)  8.7% (5.3-13.8) |

*One participant had an inconclusive TPHA

**62 participants completed rapid plasma reagin (RPR) testing and had data available for weighting

**Supplementary Table 2.** Characteristics of participants who did and did not participate in a population-based general health screening fair in a rural community in southwestern, Uganda.

| Characteristic | Total participant  number | Total population | | Participants who attended health screening | | Participants who did not attended health screening | | p-value |
| --- | --- | --- | --- | --- | --- | --- | --- | --- |
|  |  | N  Median | (%)  [IQR] | N  Median | (%)  [IQR] | N  Median | (%)  [IQR] |  |
| Age | 1606 | 37 | 26, 50 | 41 | 30, 52 | 32 | 24, 47 | <0.001 |
| Sex  Women  Men | 1630 | 912  718 | 56  44 | 473  276 | 63  37 | 439  442 | 50  50 | <0.001 |
| Relationship status  Married or cohabitating  Separated or divorced  Single, never married | 1629 | 994  289  346 | 61  18  21 | 516  152  80 | 69  20  11 | 478  137  266 | 54  16  30 | <0.001 |
| Education  None  Primary school  ≥Secondary school | 1630 | 212  837  581 | 13  51  36 | 108  449  192 | 14  60  26 | 104  388  389 | 12  44  44 | <0.001 |
| Household asset wealth  Poorest  Poorer  Middle  Less poor  Least poor | 1630 | 326  326  326  326  326 | 20  20  20  20  20 | 167  167  149  147  119 | 22  22  20  20  16 | 159  159  177  179  207 | 18  18  20  20  24 | 0.001 |
| Self-reported overall health  Very bad  Bad  Good  Very good | 1627 | 15  295  1055  262 | 1  18  65  16 | 2  167  482  97 | 0  22  64  13 | 13  128  573  165 | 1  15  65  19 | <0.001 |
| Circumcised (men only)  Not circumcised  Circumcised | 543 | 396  147 | 73  27 | 188  50 | 79  21 | 208  97 | 68  32 | 0.03 |
| HIV serostatus  Not living with HIV  Living with HIV | 1518 | 1351  167 | 89  11 | 643  80 | 89  11 | 708  87 | 89  11 | 1.00 |
| New HIV diagnosis  No new HIV diagnosis  New HIV diagnosis | 720 | 710  10 | 99  1 | 623  10 | 98  2 | -  - | -  - |  |
| Threats of or experienced intimate partner violence (IPV) in the past 3 months  No experienced IPV  Experienced IPV | 463 | 302  161 | 65  35 | 128  88 | 59  41 | 174  73 | 70  30 | 0.01 |
| Last drank alcohol  Never  Within the past 12m  >12m | 1624 | 758  492  374 | 47  30  23 | 347  218  182 | 47  29  24 | 411  274  192 | 47  31  22 | 0.44 |

**Supplementary Table 3.** Characteristics of participants who did and did not participate in a population-based syphilis screening among those who participated in a general health screening fair in a rural community in southwestern, Uganda.

| Characteristic | Total participant  number | Total population | | Health screening attendees who screened for syphilis | | Health screening attendees who did not screen for syphilis | | p-value |
| --- | --- | --- | --- | --- | --- | --- | --- | --- |
|  |  | N  Median | (%)  [IQR] | N  Median | (%)  [IQR] | N  Median | (%)  [IQR] |  |
| Age | 737 | 41 | 30, 52 | 41 | 30, 52 | 46 | 29, 61 | 0.36 |
| Sex  Women  Men | 749 | 473  276 | 63  37 | 456  268 | 63  37 | 17  8 | 68  32 | 0.68 |
| Relationship status  Married or cohabitating  Separated or divorced  Single, never married | 748 | 516  152  80 | 69  20  11 | 502  143  78 | 69  20  11 | 14  9  2 | 56  36  8 | 0.17 |
| Education  None  Primary school  ≥Secondary school | 749 | 108  449  192 | 14  60  26 | 100  438  186 | 14  60  26 | 8  11  6 | 32  44  24 | 0.05 |
| Household asset wealth  Poorest  Poorer  Middle  Less poor  Least poor | 749 | 167  167  149  147  119 | 22  22  20  20  16 | 162  160  144  142  116 | 22  22  20  20  16 | 5  7  5  5  3 | 20  28  20  20  12 | 0.96 |
| Self-reported overall health  Very bad  Bad  Good  Very good | 748 | 2  167  482  97 | 0  22  64  13 | 2  158  468  95 | 0  22  65  13 | 0  9  14  2 | 1  36  56  8 | 0.307 |
| Circumcised (men only)  Not circumcised  Circumcised | 238 | 188  50 | 79  21 | 182  49 | 79  21 | 6  1 | 86  14 | 1.00 |
| HIV serostatus  Not living with HIV  Living with HIV | 723 | 643  80 | 89  11 | 621  79 | 89  11 | 22  1 | 96  4 | 0.50 |
| New HIV diagnosis  No new HIV diagnosis  New HIV diagnosis | 633 | 623  10 | 98  2 | 616  10 | 98  2 | 7  0 | 100  0 | 1.00 |
| Current STI symptoms  No STI symptoms  STI symptoms | 749 | 448  301 | 60  40 | 429  295 | 59  41 | 19  6 | 76  24 | 1.00 |
| Lifetime STI experience  No prior lifetime STI  Prior lifetime STI | 727 | 503  224 | 69  31 | 489  221 | 69  31 | 14  3 | 82  18 | 0.30 |
| Number of sexual partners in the past 1 month | 734 | 1 | 1, 1 | 1 | 1, 1 | 1 | 0, 1 | 0.64 |
| Condom use at last sex  No condom use  Condom use | 608 | 554  54 | 91  9 | 541  53 | 91  9 | 13  1 | 93  7 | 1.00 |
| Transactional sex in the past 12 months  No transactional sex  Transactional sex | 710 | 635  75 | 89  11 | 619  73 | 89  11 | 16  2 | 89  11 | 1.00 |
| Threats of or experienced intimate partner violence (IPV) in the past 3 months  No experienced IPV  Experienced IPV | 216 | 128  88 | 60  41 | 126  86 | 60  41 | 2  2 | 50  50 | 1.00 |
| Last drank alcohol  Never  Within the past 12m  >12m | 747 | 347  218  182 | 47  29  24 | 336  210  176 | 47  29  24 | 11  8  6 | 44  32  24 | 0.97 |
